# Arginase-II promotes melanoma and lung cancer cell growth by regulating Sirt3-mtROS axis

**DOI:** 10.3389/fcell.2025.1528972

**Published:** 2025-03-19

**Authors:** Yang Yang, Andrea Brenna, Duilio M. Potenza, Santhoshkumar Sundaramoorthy, Xin Cheng, Xiu-Fen Ming, Zhihong Yang

**Affiliations:** Department of Endocrinology, Metabolism, and Cardiovascular System, Laboratory of Cardiovascular and Aging Research, Faculty of Science and Medicine, University of Fribourg, Fribourg, Switzerland

**Keywords:** arginase-II, DNA damage, melanoma, mtROS, nuclear deformation, sirt3

## Abstract

**Background:**

Aberrant mitochondrial metabolism is a key source of massive mitochondrial reactive oxygen species (mtROS) in tumour cells. Arginase-II (Arg-II), a widely expressed mitochondrial metabolic enzyme, has recently been shown to enhance mtROS production and melanoma progression. However, how Arg-II enhances mtROS and whether mtROS is involved in stimulation of cancer cell proliferation and migration remain unclear.

**Methods and results:**

Here, we show that ablation of *arg-ii* suppresses cell growth, migration, nuclear deformation, and DNA damage in melanoma cells. Vice versa, overexpression of *arg-ii* in melanoma cells promotes melanoma cell growth and migration accompanied by enhanced nuclear deformation and DNA damage. Ablation or overexpression of *arg-ii* reduces or enhances mtROS, respectively, accounting for the effects of Arg-II on melanoma growth, migration, and DNA damage. Further data demonstrate that Arg-II enhances mtROS through decreasing Sirtuin 3 (Sirt3) levels. Silencing *sirt3* promotes melanoma growth, migration, nuclear deformation, and DNA damage through enhancing mtROS. In supporting of these findings, overexpression of *sirt3* prevented Arg-II-induced mtROS production with concomitant prevention of Arg-II-induced cell growth, migration, nuclear deformation and DNA damage. Furthermore, we show that upregulation of Arg-II under hypoxia induces nuclear deformation and DNA damage through suppressing Sirt3. Similar results are obtained in A549 human lung carcinoma cells. In addition, analysis of publicly accessible datasets reveals that elevated *arg-ii* mRNA levels in human tumor samples including skin cutaneous melanoma and lung cancers associate with poorer prognosis.

**Conclusion:**

Altogether, our findings demonstrate a critical role of Arg-II-Sirt3-mtROS cascade in promoting melanoma growth, migration, nuclear deformation, and DNA damage linking to melanoma progression and malignancy, which could be therapeutic targets for cancers such as melanoma and lung carcinoma.

## Introduction

As one of the most aggressive and therapy-resistant cancers, melanoma represents an important challenge in cancer therapy and public health worldwide (Arnold et al., 2022), despite significant advancements in approved clinical treatment for melanoma including surgical resection, adjuvant therapy after surgical resection, chemotherapy, radiotherapy, cytokine therapy, targeted therapy and immunotherapy (Rahimi et al., 2023; Curti and Faries, 2021). Advanced melanoma is often hard to cure with current treatments (Ernst and Giubellino, 2022). Identification of novel molecular mechanisms underlying melanoma progression towards developing alternative therapeutic target (s) for advanced melanoma patients who have already tried and failed other drugs is an unmet need.

Arginase-II (Arg-II) is a widely expressed extrahepatic mitochondrial enzyme that catabolizes L-arginine to L-ornithine and urea and is overexpressed in a variety of cancers, such as cancers of kidney, pancreas, breast, colorectal, lung and melanoma (Niu et al., 2022). A role of Arg-II in progression of various cancers has been demonstrated by many studies including our own (Niu et al., 2022; Grzybowski et al., 2022; Zaytouni et al., 2017; Yu et al., 2020; Roci et al., 2019). Silencing or loss of *arg-ii* significantly inhibits the growth of pancreatic cancer (Zaytouni et al., 2017), while the growth of colorectal cancer and kidney cancer cells was inhibited by arginase inhibitor (Grzybowski et al., 2022). Previous studies showed that Arg-II promotes proliferation and migration of melanoma cells (Yu et al., 2020), is able to enhance mitochondrial reactive oxygen species (mtROS) production in many types of cells (Ming et al., 2012; Ren et al., 2022), and mtROS has been shown to play a critical role in tumorigenesis and malignancy (Idelchik et al., 2017). It is however not known, how Arg-II promotes mtROS production and whether mtROS mediates Arg-II’s effect in promoting tumour growth and metastasis-related process such as migration.

Evidence has been presented that Sirtuin 3 (Sirt3), a NAD^+^-dependent protein deacetylase that is localized in mitochondria (Giralt and Villarroya, 2012), exerts a wide range of biological functions and have emerged as an important player in aging, metabolic diseases, cardiovascular disease, neurodegenerative disease, and cancer development (Ansari et al., 2017; Torrens-Mas et al., 2017). Sirt3 has been recognized as a tumour suppressor and inhibits tumour growth and carcinogenesis by reducing mtROS production (Bell et al., 2011). In general, mitochondria in cancer cells are characterized by the overproduction of mtROS, contributing to tumorigenicity and metastasis by induction of genomic instability (Yang et al., 2016). Moreover, nuclear deformation has been linked to cancer malignancy by causing DNA damage and promoting cancer cells migration that requires cells to deform their nucleus to squeeze through the available spaces (Kalukula et al., 2022). However, whether Arg-II, Sirt3 and mtROS form a network affecting melanoma as well as other tumour cell growth and malignancy has not been studied.

In the current study, using *arg-ii* ^−/−^ as well as the *arg-ii*-overexpressing melanoma and lung carcinoma cells, we demonstrate a critical role of Arg-II-Sirt3-mtROS cascade in promoting tumour cell growth, migration, nuclear deformation and DNA damage linking to progression and malignancy of melanoma as well as of lung cancer cells, which could be the therapeutic targets for treatment the cancers.

## Materials and methods

### Reagent and antibody

Reagents were purchased from the following sources: The primary rabbit antibodies against Arg-II (#55003), Sirt3 (#5490) and eNOS (#9571S) were purchased from Cell Signaling Technology (Allschwil, Swizerland); rabbit anti-iNOS (ab178945), nNOS (ab76067) and p-γH2A.X (phos-S139) antibody (ab81299) was purchased from Abcam (Cambridge, UK); The primary mouse antibody against Sirt1 (ab110304) were purchased from Abcam (Cambridge, UK), the mouse antibody against Arg-I (sc-18351) was obtained from Santa-Cruz (Schaffhausen, Switzerland), the mouse anti-tubulin (Art.T5168) was from Sigma-Aldrich (Schaffhausen, Switzerland); Alexa Fluor® 594 conjugated-goat anti-rabbit IgG (H + L) secondary antibody (A-11012) and goat anti-mouse IgG (H + L) (A-21057) as well as MitoSOX (M36008) and Wheat Germ Agglutinin (WGA) (W11261) were purchased from ThermoFisher Scientific (Basel, Switzerland). Mito-TEMPO (SML0737) and crystal violet (C0775) were purchased from Sigma-Aldrich (Schaffhausen, Switzerland). Sirt3 siRNA (SC61555), and control siRNA (SC36869) were purchased from Santa Cruz (Heidelberg, Germany). Sirt3 Flag plasmid (pCDNA3.1+/CMV-Sirt3-Flag) was purchased from Addgene (13,814). Trans-well Insert chambers with 8.0 µm Coloured PET Membrane (351,152) were bought from Corning (Arizona, United States).

### Cell culture

The Me276 cells (a human melanoma cell line derived from lymph node of a patient with melanoma metastasis (Caballero et al., 2010)) and A549 cells [adenocarcinoma human alveolar basal epithelial cells (Giard et al., 1973)] was purchased from ATCC and were maintained in RPMI1640 (PAN-Biotech, Aidenbach, Germany), supplemented with 5% heat-inactivated foetal bovine serum (FBS) (Gibco), 100 U/mL penicillin and 100 μg/mL streptomycin (Gibco, Zug, Switzerland). Cells were cultured at 37°C in a humidified incubator containing 5% CO_2_ and the culture media was changed every 2 days. Hypoxic conditions were achieved by placing the cultures dishes or plates in a Coy *In Vitro* Hypoxic Cabinet System (The Coy Laboratory Products, Grass Lake, MI USA) at 1% O_2_ with 5% CO_2_ and N_2_ as balance (Liang et al., 2019).

### Generation of stable *arg-ii* ^*−/−*^ cancer cell lines using CRISPR/Cas9 gene editing technology

*arg-ii* ^−/−^ A549 cells were generated using CRISP/Cas9 technologies as described (Zhu et al., 2023). *arg-ii* ^−/−^ Me276 cells were generated using the same method. Briefly, sgRNA targeting human *arg-ii* (the top strand of the sgRNA that recognizes the target DNA region of human *arg-ii*: GGGACTAACCTATCGAGA) was cloned into pSpCas9(BB)-2A-Puro (PX459) V2.0 (Plasmid #62988, Addgene) to generate pSpCas9(BB)-2A-Puro (PX459)-U6/sgRNA-h*arg-ii*. Transfection of pSpCas9(BB)-2A-Puro (PX459)-U6/sgRNA-h*arg-ii* was performed using Lipofectamine™ 3,000 Transfection Reagent (L3000008, Invitrogen™) according to the manufacturer’s protocol. Briefly, Me276 cells were plated in 6-cm dish at a density of 1 × 10^6^ cells 24 h before transfection. Per 1 × 10^6^ cells, diluted plasmid DNA (5 μg, diluted with P3000™ Reagent) and diluted Lipofectamine™ 3,000 Transfection Reagent were mixed at a 1:1 ratio and incubated at room temperature for 15 min. The DNA-lipid complex was then added to the cells. To select the sgRNA-positive cells, 48 h post transfection, cells were treated with puromycin (1 μg/mL) for 72 h until all non-transfected cells died. Puromycin-resistant cells were then allowed to recover in medium without puromycin for 1 week before seeding single cells into 96-well plate by dilution method. Single clones were then expanded and screened for *arg-ii* ^−/−^ by immunoblotting.

### Generation and transduction of recombinant adenoviral constructs

Recombinant adenovirus (rAd) expressing *arg-ii* driven by CMV promoter (rAd/CMV-*arg-ii*) and the relevant control vector (rAd/CMV-*lacz*) were produced as previously described (Yepuri et al., 2012). To overexpress *arg-ii*, cells were seeded in 6-well or 12-well plate at a density of 3*10^5^/mL or 5*10^4^/mL, respectively. After overnight culture, cells were transduced with rAd/CMV-*lacz* as control or rAd/CMV- *arg-ii* at the titers of 100 MOI (multiplicity of infection) and cultured in complete medium for 48 h before the experiments were performed.

### Transient transfection of small interfering RNA (siRNA) or plasmid

Melanoma cells were seeded in 6-well or 12-well plate at a density of 3*10^5^ cells or 5*10^4^ cells, respectively. After overnight culture, siRNA targeting Sirt3 or scrambled control siRNA, pcDNA3.1+/CMV-Sirt3-Flag or empty control plasmid pcDNA3.1+/CMV were transfected by using Lipofectamine 3,000 (Thermo Fisher, Rockford, IL, United States) according to the manufacturer’s instructions. 48 h post transfection, experiments were performed. Gene silencing efficacy as well as overexpression was assessed by immunoblotting analysis.

### Colony-forming assay

The wild type (*wt*) and *arg-ii* ^−/−^ Me276 or A549 cells were seeded at 500 cells/well in 6-well culture plate in duplicate. For *arg-ii*-overexpression experiments, 200 cells/well (6-well) were seeded 48 h post transduction. After seeding, the cells were kept at 37°C in 5% CO_2_ incubator for 12 days and culture media were changed every 3 days. After 12 days, cells were washed with 1x PBS and then fixed with 10% Neutral Buffered Formalin (NBF) buffer for 15 min, followed by staining with 0.5% crystal violet buffer for 15 min. The images of total colonies in each well were taken by Canon G16 camera. The number of visible colonies after staining with crystal violet was counted manually after circling individual colonies.

Since some very big colonies may be resulted from a merge of more than one colony, the total area of colonies in a plate was also quantified by ImageJ software and presented along with the colony number in the same graphics.

### Cell number counting

The cell number counting was performed 48 h post transduction. For experiment with TEMPO, cells were treated with TEMPO (10 μmol/L) 48 h post transduction for 1 h and further cultivated for 24 h in the absence of TEMPO and then subjected to collection of cell suspension by trypsinization and cell counting. Cell number was counted with the Neubauer cell counting chamber (Blaubrand) and expressed as cell number/well (Park et al., 2024).

In some figures (Figure 3C; Supplementary Figure S4B), the number of cells/mm^2^ was obtained by first counting cell number in the DAPI-stained confocal image manually, and then quantified by ImageJ software (US National Institutes of Health) (Venter and Niesler, 2019).

### Treatment of cells with TEMPO

Except for colony-formation assay, cells were treated with TEMPO (10 μmol/L) for 1 h. After removal of TEMPO, the cells were further cultivated for 24 h in the absence of TEMPO and then subjected to the relevant assays as described in individual figures. For colony-formation assay, TEMPO was added to the medium when cells were attached after seeding. Fresh media containing TEMPO (10 μmol/L) was changed every 3 days for a total period of 12 days.

### Immunoblotting

Cell lysate preparation, SDS-PAGE and immunoblotting, antibody incubation, and signal detection were performed as described previously (Ming et al., 2012). Briefly, cells were lysed in a lysis buffer containing the protease inhibitors cocktail (B14002, B15002, Biotool). After three cycles of freeze-thaw in liquid nitrogen, cell debris and nuclei were removed by centrifugation (Sorvall Legend Micro 17R) at 12,000 × *g* for 15 min at 4°C. The protein concentration of the supernatant was determined by a Lowry protein assay (500-0116, Bio-Rad). Proteins from the samples were loaded on the sodium dodecyl sulphate-polyacrylamide gel (SDS-PAGE), separated by electrophoresis, and transferred to PVDF membranes (Millipore). Ponceau staining and tubulin were used as the loading control. The resultant membranes were first blocked with skimmed milk (5%) for 1 h, incubated with the designated primary antibody (in PBS-Tween supplemented with 5% milk) overnight at 4°C. Membranes were washed with 5% milk buffer three times and further incubated with anti-mouse (Alexa fluor 680 conjugated) or anti-rabbit (IRDye 800 conjugated) secondary antibody for 1.5 h. Dilutions of each primary antibody are presented in Supplementary Table S1. The signals were detected using Odyssey Infrared Imaging System (LI-COR Biosciences). Quantification of the signals was performed using Image Studio Lite version 5.2.

### MitoSox and wheat germ agglutinin (WGA) staining

Mitochondrial superoxide generation was assessed by MitoSOX™ Red Mitochondrial Superoxide Indicator (Thermo Fisher) according to the manufacturers’ instructions. Briefly, live cells were incubated with medium containing MitoSox (5 μmol/L) for 10 min in cell culture incubator. After gentle washing 3 times with PBS (5 min each time), the cells were fixed with 10% NBF for 10 min, permeabilized with 0.1% Triton X-100 for 15 min, washed with PBS 3 times followed by counterstaining of the nuclei with DAPI for 5 min. Images were taken under the Leica TCS SP5 confocal laser microscope with ×63 objectives. The intensity of the MitoSox fluorescence signals per cell was quantified by Leica X Life Science Microscope software (Leica Microsystems).

Staining of mammalian cell plasma membranes was performed using WGA Alexa Fluor™ 488 Conjugate according to the instructions from the manufacturer (ThermoFisher). In brief, cells were incubated with diluted WGA Alexa Fluor™ 488 Conjugate (10 μg/mL) medium for 15 min in the incubator and then fixed with 10% NBF buffer for 10 min, followed by permeabilization with 0.1% triton X-100 for 15 min and counterstaining of the nuclei with DAPI for 5 min. Signals were detected and photographed by the Leica TCS SP5 confocal laser microscope with ×63 objectives.

### Assessment of nuclear deformation

Nuclei were stained with DAPI and images were captured under a Leica fluorescence microscope. Nuclear deformation was assessed by observing the morphology of the DAPI-stained nuclei. To better observe the shape of the nucleus, blue colour of DAPI was converted to white on dark background using Leica software. The percentage of normal ovoid or kidney-shaped nuclei (1 lobe) and deformed multilobed nuclei with ≥2-lobes (equal to or more than 2 lobes) in the field of view for each image was counted and calculated. Five fields of each image were analysed and the average number of cells with deformed multilobed nuclei was presented as percentage of the total cell number.

### Assessment of DNA damage by immunofluorescence staining for p-γH2AX and comet assay

Cells were grown on sterile glass slides in Petri dishes. After gently washing with PBS, cells were fixed with 10% NBF for 10 min and permeabilized with 0.1% Triton X-100 solution for 15 min. Cells were then incubated in 1% blocking solution (10% goat serum in 1% BSA solution) for 1 h at room temperature followed by incubation with anti-p-γH2AX antibody (1:100) at 4°C overnight. After three times of washing with PBS, the secondary antibody was added and incubated at room temperature for 1.5 h. After washing with PBS three times, cells were then counter-stained with DAPI for 5 min. Immunofluorescence signals were visualized under Leica TCS SP5 confocal laser microscope.

The comet assay was performed according to the manufacturer’s instructions (ab238544, Abcam Plc., Cambridge, United Kingdom). Briefly, wild-type (wt) and *arg-ii* ^−/−^ Me276 melanoma cells were cultured in RPMI-1640 medium supplemented with 10% fetal bovine serum (FBS) and 1% penicillin-streptomycin. 7,000 Me276 melanoma cells were collected for the comet assay. The cells were washed once with phosphate-buffered saline (PBS) and then mixed with Comet Agarose at a 1:10 ratio (v/v). A total of 75 μL of the cell-agarose mixture was immediately transferred onto the top of the Comet Agarose Base Layer. The cells were lysed at 4 °C, followed by electrophoresis in a cold alkaline electrophoresis buffer for 15 min at 1 V/cm. The comet slides were rinsed twice with double-distilled H_2_O for 5 min each and fixed with 70% cold ethanol for 5 min. After air-drying, the slides were stained with Vista Green DNA Dye and imaged using a fluorescence microscope. Cells positive for comet tails were quantified as DNA-damaged cells.

### The trans-well cell migration assay

5 × 10^4^ cells per well were plated in the trans-well insert (Corning® FluoroBlok™ 24-Multiwell Insert with 8.0 µm Colored PET Membrane) with complete medium with 5% FBS in both lower and upper chamber. 2 h post plating, 10% FBS was added into the lower chamber. After 48 h, inserts were transferred to a clean 24-well plate, washed with 1x PBS for 2 min and cells were fixed with 10% NBF buffer for 10 min. After two times washing with PBS (5 min per wash), cells were permeabilized with 0.1% Triton X-100 for 15 min followed by staining of the nuclei with DAPI for 5 min in darkness. The DAPI signals from cells that have migrated to the bottom of the trans-well insert were then detected by a bottom-reading fluorescence plate reader (BioTek Cytation 5). The results were analysed by Gen5 software and presented as migrated cells/mm^2^.

### Analysis of publicly accessable datasets

Gene Expression Analysis: the expression of *arg-ii* was analyzed using the open-source database GEPIA (Gene Expression Profiling Interactive Analysis), an online platform that processes RNA sequencing expression data from The Cancer Genome Atlas (TCGA) and Genotype-Tissue Expression (GTEx) databases. Box plots were generated using the platform’s differential expression analysis and profiling features. In this study, the GEPIA database (http://gepia.cancer-pku.cn/index.html) was utilized to investigate *arg-ii* gene expression across multiple cancer types (Tang et al., 2017).

Survival Analysis: to evaluate the prognostic significance of *arg-ii* expression, Kaplan-Meier survival plots were generated using data from all stage lung cancer patient samples. The analysis was performed using the KMplot online tool (https://kmplot.com/analysis/index.php?p=home), which integrates survival data from multiple cancer studies (Gyorffy, 2024a; Gyorffy, 2024b).

### Statistical analyses

In all experiments, n indicates the number of individual experiments. The Shapiro-Wilk test (that determines normality when n ≥ 3) was used first to determine whether the data deviated from Gaussian distributions. Since all data are normally distributed, parametric statistical analysis was performed. Student’s t-test for unpaired observations was applied to compare the averages of two groups and one-way analysis of variance (ANOVA) was used to analyze the difference between the means of more than two groups. All data are expressed as standard error of the mean (SEM). GraphPad Prism software version 9.0 was used for the statistical analysis. Differences were considered statistically significant at *p* < 0.05.

## Results

### Arg-II promotes colony formation and migration of melanoma and lung cancer cells

**FIGURE 1:**
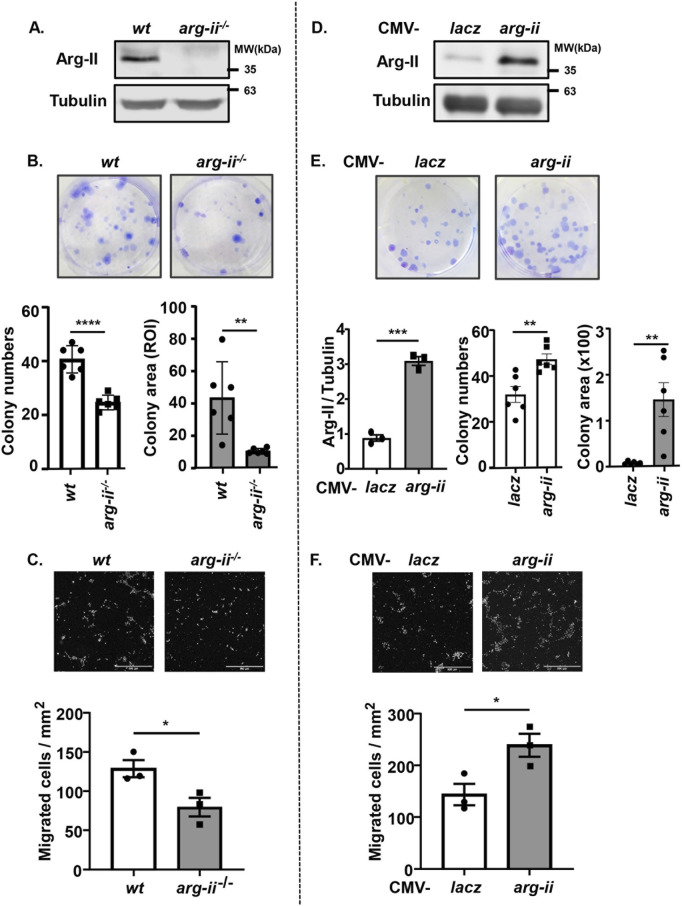
Arg-II promotes colony formation and migration of Me276 melanoma cells. **(A–C)** Cultured *wt* or *arg-ii−/−* Me276 cells were subjected to **(A)** cell lysate extraction and immunoblotting analysis of Arg-II, **(B)** colony formation assay after 12 days cultivation, seeded at 500 cells/well on day 0 (n = 6, ROI: region of interest), or **(C)** trans-well migration assay (n = 3). **(D–F)** wt Me276 melanoma cells were transduced with rAd/CMV-lacz (lacz) as control or -arg-ii to overexpress Arg-II (arg-ii). Cells were subjected to **(D)** immunoblotting analysis (n = 3) 48 h post-transduction. **(E)** colony formation assay 12 days post-transduction, seeded at 200 cells/well on day 0 (n = 6), or **(F)** trans-well migration assay 48 h post-transduction (n = 3). Scale bar: 500 µm. Tubulin serves as a loading control for immunoblotting analysis. The graphics present the quantification of the results shown in the corresponding upper images. *p < 0.05, **p < 0.01, ***p < 0.005, ****p < 0.0001 between the indicated groups.

To determine the effects of Arg-II on tumour cell colony formation which is an important process for cancer progression, human melanoma cell line Me276 lacking *arg-ii* ^*−/−*^ (*arg-ii* ^*−/−*^) was generated by CRISPR/Cas9 technology. Immunoblotting confirmed the Arg-II deficiency in the cells (Figure 1A). Twelve days after cell seeding in culture, *arg-ii* ^*−/−*^ Me276 cells showed fewer colonies and smaller colony area as compared to the *wt* control cells (Figure 1B). This result is consistent with previous data showing a reduced cell number in cells transduced with rAd/U6-*arg-ii* (to silence *arg-ii*) (Yu et al., 2020). Moreover, Me276 cell migration activity as examined by trans-well migration assay was significantly reduced by *arg-ii* knockout (*arg-ii* ^*−/−*^) (Figure 1C). To further confirm the role of Arg-II in colony formation and migration of the melanoma cells, *arg-ii* gene was overexpressed in the Me276 cells by adenoviral transduction, which was verified by immunoblotting 48 h post transduction (Figure 1D). In agreement with *arg-ii* knockout experiments, *arg-ii* overexpressing Me276 cells formed more colonies and bigger colony area (Figure 1E) and exhibited enhanced migration activity (Figure 1F) as compared to the *wt* control cells. These results demonstrate an important role of Arg-II in promoting melanoma cell growth and migration. Similar results were also demonstrated with another cancer cell line, i.e., the lung carcinoma cell A549 (Supplementary Figure S1).

### Arg-II promotes nuclear deformation and DNA damage in melanoma and lung cancer cells

**FIGURE 2:**
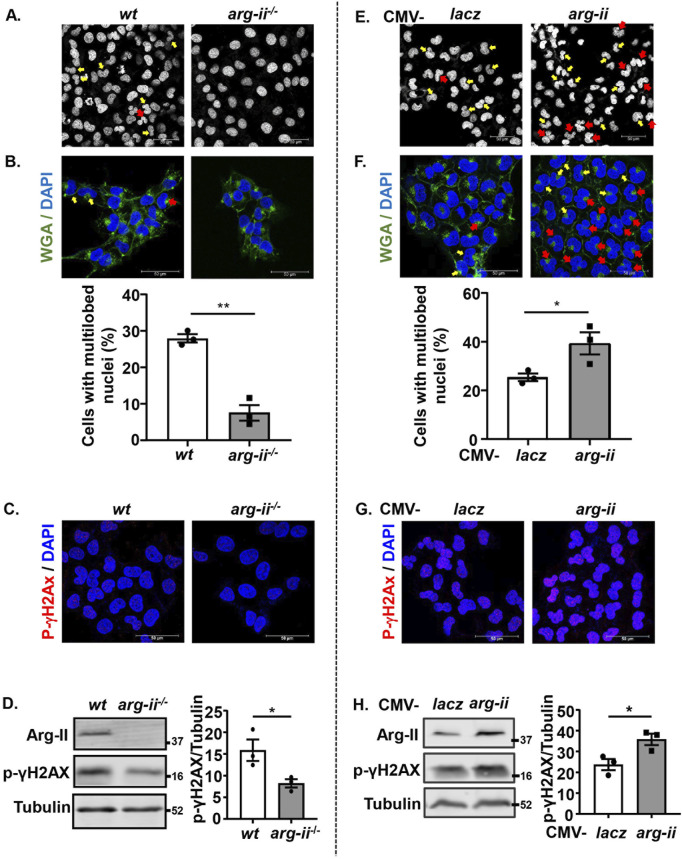
Arg-II promotes nuclear deformation and DNA damage in melanoma cells. Experiments were performed as described in Figure 1, except that DAPI and WGA staining were performed. **(A, E)** Confocal microscopic imaging of DAPI (white) staining. **(B, F)** WGA (green) and DAPI (blue) staining. Quantification of number of cells with multi-lobed nuclei (presented as %) shown in A (n = 3) or E (n = 3), respectively. Yellow arrows indicate 2-lobed nuclei and red arrows indicate 2+-lobed nuclei. **(C–H)** Experiments were performed as described in Figures 2A, E, except that the cells were subjected to immunostaining or Immunoblotting using anti-p-γH2AX-S139 antibody. **(C, G)** Immunostaining for p-γH2AX-S139 (red) followed by DAPI (blue) staining. **(D, H)** Immunoblotting analysis of p-γH2AX-S139, Arg-II and Tubulin. Quantification of the signals is presented in the graphics next to the corresponding blot. n = 3. Scale bar: 50 µm. *p < 0.05, **p < 0.01 between the indicated groups.

We further investigated the effects of *arg-ii*-knockout on nuclear morphology and DNA damage on the tumour cells in view that these are indicative of tumour metastasis and malignancy. A decrease in number of cells with multi-lobed nuclei was observed in *arg-ii* ^−/−^ melanoma cells when compared with the *wt* cells (Figure 2A). To further confirm that Arg-II induces nuclear deformation and to better demonstrate whether the heteromorphic nuclei are in individual cells, cells were stained with DAPI and WGA to visualize nuclei and cell border, respectively (Figure 2B). For evaluation, the percentage of ovoid or kidney-shaped (1-lobed) nuclei and heteromorphic multi-lobed nuclei in the field of view for each image was counted and calculated. *Arg-ii* ^*−/−*^ cells revealed significantly lower percentage of cells with heteromorphic multi-lobed nuclei (Figures 2A, B). In line with these results, *arg-ii*-overexpressing cells showed more multi-lobed nuclei (Figures 2E, F). Of note, *arg-ii*-overexpression caused more severe nuclear deformation as evidenced by more cells with 3- and 4-lobed nuclei (Figure 2F), while *wt* cells show mostly only 2-lobed nuclei (Figure 2B).

Along with these results, phosphorylated histone γ-H2AX on serine 139 (p-γH2AX-S139), a sensitive molecular marker of DNA damage, was detected at baseline levels in *wt* cells and decreased in *arg-ii* ^−/−^ melanoma cells (Figures 2C, D). Moreover, the decreased DNA damage in *arg-ii* ^−/−^ melanoma cells was also evident by comet assay revealing a decresed percentage of comet tail-positive cells (Supplementary Figure S2). In line with these data, the levels of p-γH2AX-S139 were enhanced in melanoma cells overexpressing *arg-ii* (Figures 2G, H). Similar results were also obtained in A549 cells (Supplementary Figure S3). These results demonstrate that the Arg-II-induced nuclear deformation is linked to increase in migration and DNA damage, which increases the cancer cell malignancy.

### Role of mtROS in Arg-II’s effect in melanoma and lung cancer cells

Next, we wish to investigate how Arg-II affects tumour growth and malignancy. Since one of the best characterized biological functions of Arg-II is attributable to its regulation of nitric oxide synthase (NOS)-NO pathway, we examined the expression of eNOS, iNOS and nNOS as well as another isoform of arginase (Arg-I) in melanoma and lung cancer cells. Neither eNOS, iNOS, nNOS nor Arg-I could be detected by immunoblotting in the human melanoma or A549 lung cancer cells (data not shown). This result implies that the effects of Arg-II on melanoma or lung cancer cell growth and malignancy are independent of Arg-I or NOS-NO pathway.

**FIGURE 3:**
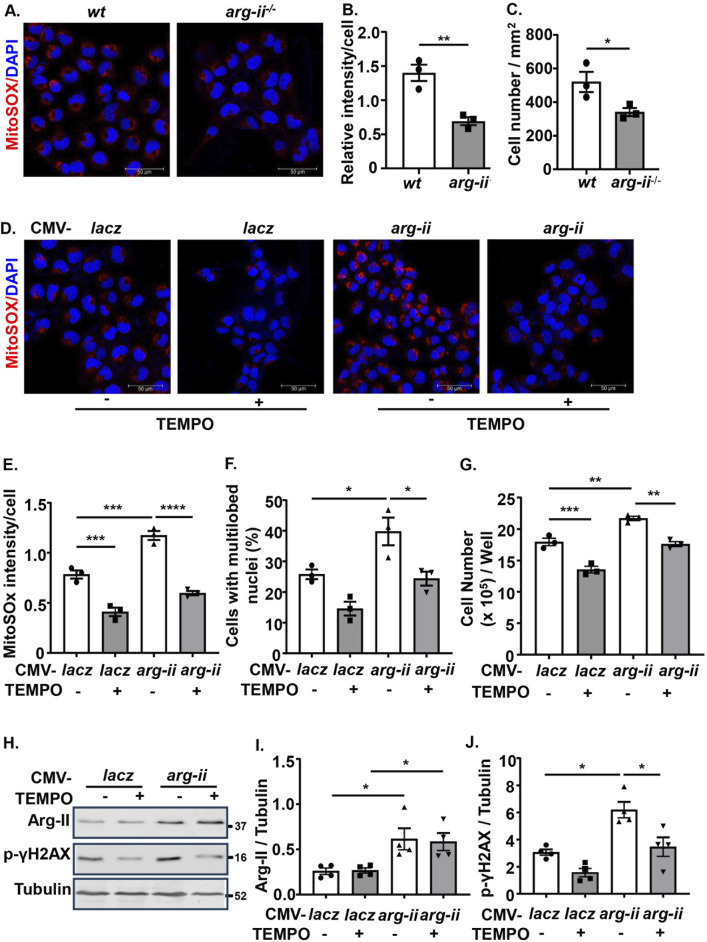
Arg-II promotes melanoma cell growth, nuclear deformation and DNA damage through mtROS. **(A)** Cultured *wt* and *arg-ii* ^*−/−*^ Me276 cells were subjected to MitoSox staining for mtROS (red) followed by counterstaining of nuclei with DAPI (blue). **(B)** Quantification of MitoSox signals in A. **(C)** Cell numbers was counted from the immunofluorescence images and presented as cell number/mm^2^ of image shown in A (n = 3). **(D)** *wt* Me276 cells were transduced with rAd-CMV-*lacz* (Con) or -*arg-ii (arg-ii).* 48 h post transduction, cells were treated with TEMPO (10 umol/L) for 1 h. After removal of TEMPO, the cells were further cultivated for 24 h and then subjected to MitoSox staining followed by DAPI staining. **(E, F)** Quantification of MitoSox signals **(E)** and % of cells with multilobed nuclei **(F)** from the images shown in **(D)**. **(G)** Cell number per well counted with the Neubauer cell counting chamber and presented as cell number/well. **(H)** Experiments were performed as described in Figure 3D except that immunoblotting analysis was performed. **(I, J)** Quantification of Arg-II **(I)** and p-γH2AX-S139 **(J)** from the blots shown in **(H)**. Scale bar: 50 µm. *p < 0.05, **p < 0.01, ***p < 0.001, ****p < 0.0001 between the indicated groups.

**FIGURE 4:**
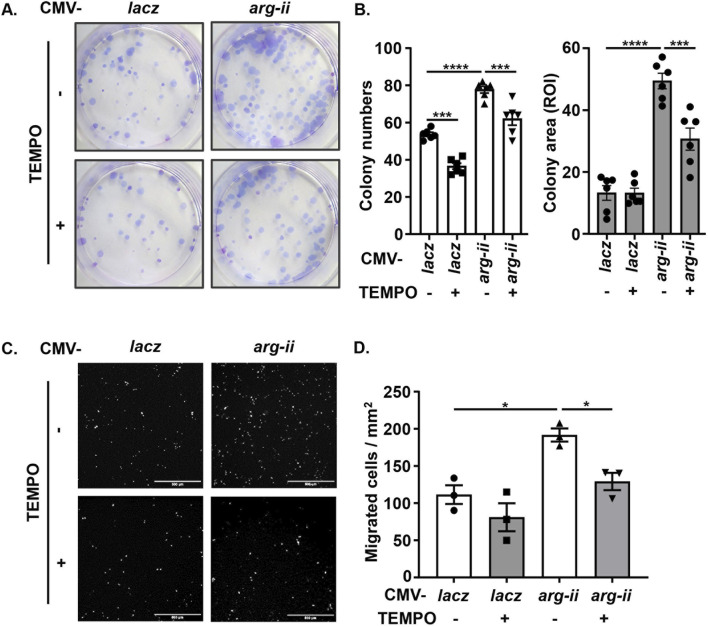
Arg-II promotes tumor colony formation and migration via mtROS. Experiments were performed as described in Figure 3D except that **(A, B)** colony formation assay (ROI: region of interest) and **(C, D)** trans-well migration assay were performed 12 days or 48 h, respectively, post transduction, Scale bar: 500 µm. *p < 0.05, ***p < 0.001, ****p < 0.0001 between the indicated groups.

Since previous studies provide evidence that mtROS generation is enhanced by Arg-II in various cell types (Ming et al., 2012; Ren et al., 2022), the role of mtROS in Arg-II-mediated effects on the cancer cells were then investigated. Indeed, the production of mtROS (mitochondrial superoxide) in the melanoma cells as demonstrated by MitoSOX staining, was significantly reduced in *arg-ii* ^*−/−*^ cells as compared to the *wt* melanoma cells (Figures 3A, B), which was accompanied by decrease in cell number (Figure 3C). Conversely, overexpression of *arg-ii* enhanced mtROS levels and the cell number, which was inhibited by MitoTEMPO (10 μmol/L), a mitochondria-targeted antioxidant and a specific scavenger of mitochondrial superoxide (Figures 3D–G). Moreover, the multi-lobed nuclei formation (Figures 3D, F) and DNA damage (Figures 3H, J) as well as colony formation and migration of the cells overexpressing *arg-ii* (Figure 4) all were inhibited by MitoTEMPO. Of note that MitoTEMPO did not affect Arg-II levels (Figure 3I). These results were also observed in the lung cancer cells (Supplementary Figures S4, S5). Together, these results demonstrate that Arg-II promotes melanoma and lung cancer cell growth and malignancy through enhanced mtROS production.

### Arg-II suppresses Sirt3 expression in melanoma and lung cancer cells

**FIGURE 5:**
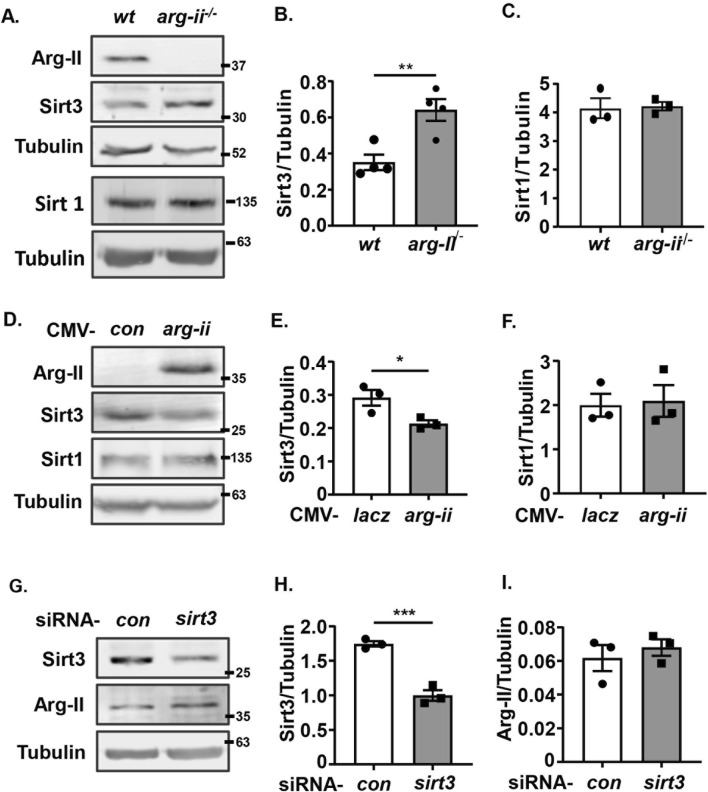
Arg-II suppresses Sirt3 expression. Immunoblotting analysis of Arg-II, Sirt3, Sirt1 with lysates prepared from **(A–C)** *wt* and *arg-ii* ^*−/−*^ Me276 cells (n = 4), **(D–F)** *arg-ii* ^*−/−*^ Me276 cells transduced with rAd-CMV-*lacz* (Con) or -*arg-ii* (*arg-ii*), **(G–I)** *wt* Me276 cells transfected with scrambled control siRNA or siRNA-*sirt3* for 48 h (n = 4). Tubulin served as loading control. The graphics on the right present the quantification of the results shown in the corresponding left panels. *p < 0.05, *p < 0.01, ***p < 0.001 between the indicated groups.

Sirt3, a mitochondrial sirtuin, has been reported to exert antioxidant and anti-cancer effects (Bell et al., 2011). In accordance with its anti-cancer effects, Sirt3 was enhanced in *arg-ii* ^*−/−*^ melanoma cells (Figures 5A, B). Conversely, overexpression of *arg-ii* decreased Sirt3 levels (Figures 5D, E). In contrast to Sirt3, Sirt1 was not affected either by *arg-ii* knockout or by *arg-ii* overexpression (Figures 5A, C, D, F). It’s to note that silencing *sirt3* (Figures 5G, H) did not significantly affect Arg-II protein levels (Figures 5G, I), suggesting Arg-II is the upstream of Sirt3. Similar results were obtained in A549 lung cancer cells (Supplementary Figure S6). These results demonstrate that Arg-II is the upstream of Sir3 and suppresses Sirt3 expression.

### Sirt3 inhibits ROS generation, growth and malignancy of melanoma and lung cancer cells

**FIGURE 6:**
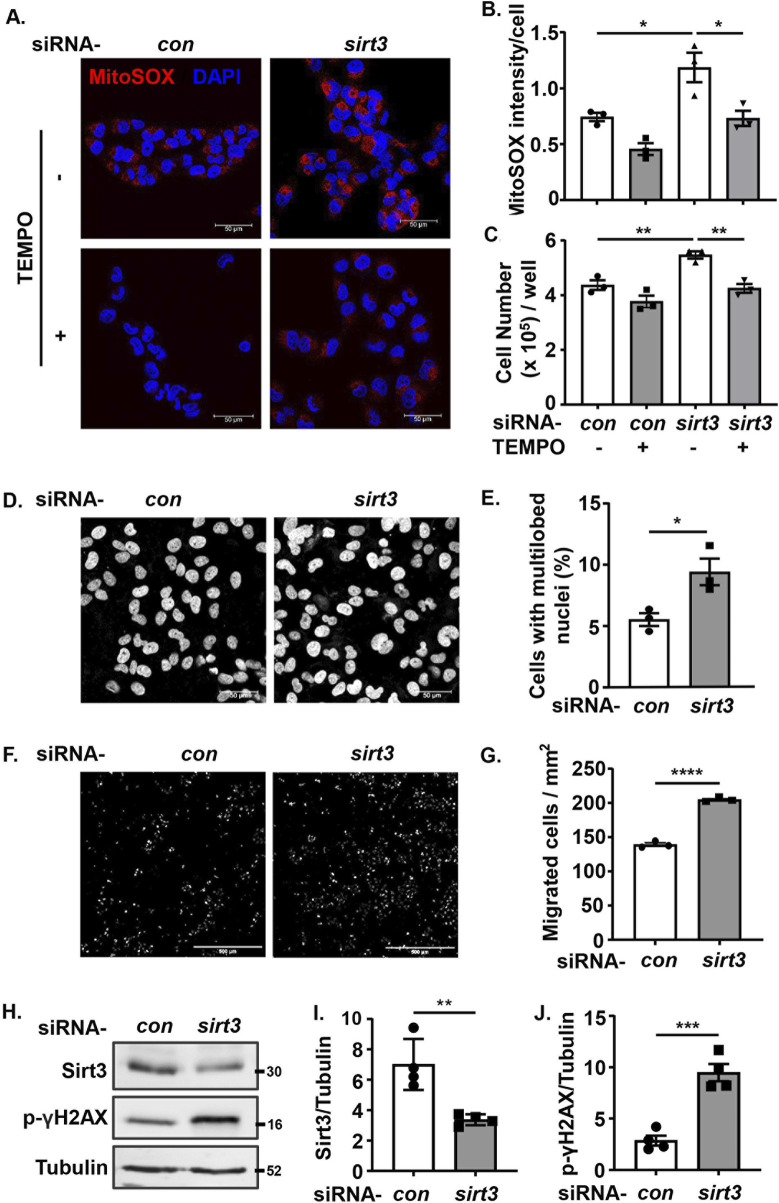
Silencing Sirt3 promotes melanoma growth and malignancy by enhancing mtROS. *wt* Me276 cells were transfected with scrambled control siRNA or siRNA-*arg-ii*, 24 h post-transfection, TEMPO (10 umol/L) was added to the cells and removed after 1 h. The cells were further cultivated for another 24 h, and then subjected to following analysis. **(A)** MitoSox staining (red). Scale bar: 50 µm. **(B)** Quantification of the mtROS signals shown in A (n = 3). **(C)** Cell number per well counted with the Neubauer cell counting chamber and presented as cell number/well (n = 6). **(D, E)** DAPI staining (white) to assess the nuclei deformation. Scale bar: 50 µm. **(F, G)** trans-well migration assay. Scale bar: 500 µm. **(H–J)** immunoblotting analysis of Sirt3 and p-γH2AX-S139. Tubulin served as loading control. The graphics on the right present quantification of the signals in the corresponding images or blots. *p < 0.05, **p < 0.01, ***p < 0.001, ****p < 0.0001 between the indicated groups.

In supporting the recognized anti-oxidant and anti-cancer effects of Sirt3, silencing *sirt3* in *wt* melanoma cells enhanced mtROS production along with increased melanoma cell growth, which was prevented by inhibition of mtROS with MitoTEMPO (Figures 6A–C). These results confirm that Sirt3 inhibits cell growth by preventing mtROS generation. In parallel, silencing of Sirt3 in the melanoma cells resulted in a significant nuclear deformation (Figures 6D, E), an increase in migration (Figures 6F, G) and DNA damage (Figures 6H–J). Of note, mtROS didn’t affect Arg-II-induced suppression of Sirt3 (Supplementary Figure S7).

### Arg-II induces mtROS generation, cell growth and malignancy through suppressing Sirt3

**FIGURE 7:**
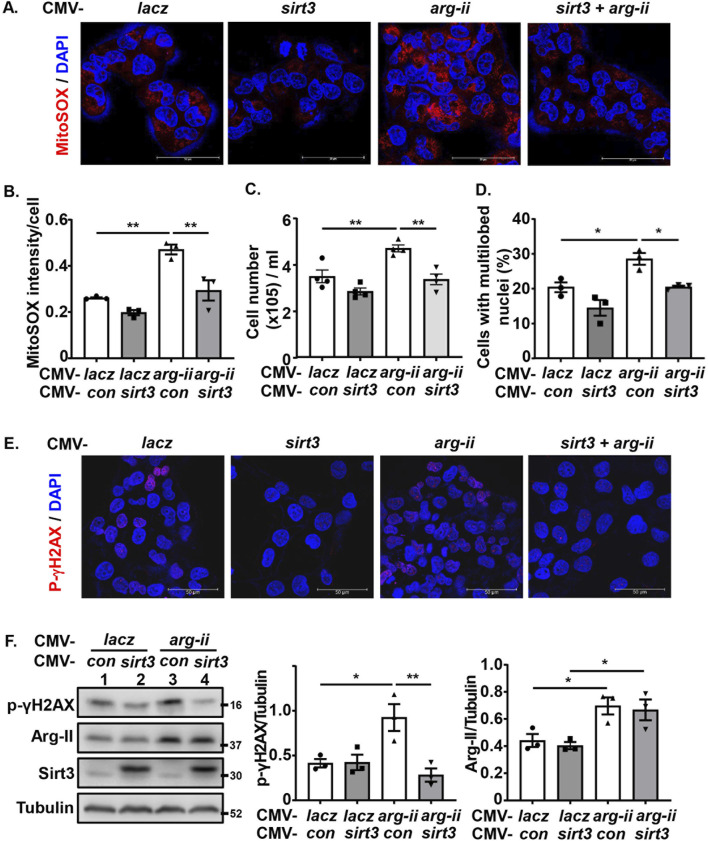
Arg-II induces mtROS generation, cell growth and malignancy through suppressing Sirt3. *wt* Me276 cells were transfected with plasmid CMV-control or -*sirt3* to overexpress Sirt3. 24 h post-transfection, cells were transduced with rAd-CMV-*lacz* (used as control) or -*arg-ii*. 48 h post transduction, following analysis was performed. **(A, B)** MitoSox (red) and DAPI (blue) staining. **(C)** Cell number/well counted with the Neubauer cell counting chamber. **(D)** From the images shown in 7A, **(C)** and % of the cells with multi-lobed nuclei **(D)** were counted. **(E)** DNA damage assessed by immunostaining for p-γH2AX-S139 (red) followed by DAPI staining (blue). **(F)** Immunoblotting analysis of p-γH2AX-S139, Arg-II and Sirt3. Tubulin served as loading control. Scale bar: 50 µm. *p < 0.05, **p < 0.01, ***p < 0.001 between the indicated groups.

**FIGURE 8:**
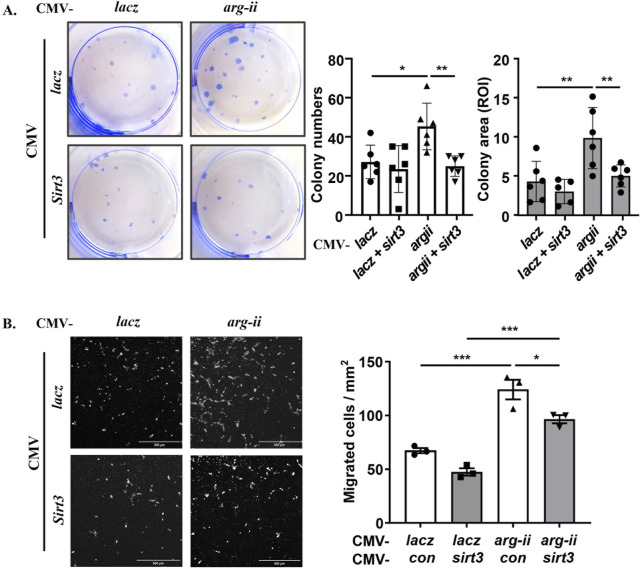
Arg-II enhances colony formation and migration through suppressing Sirt3. *wt* Me276 cells were transfected as described in Figure 7. **(A)** Colony formation assay performed after 12 days cultivation (ROI: region of interest). **(B)** trans-well migration assay performed 48 h post transduction. Scale bar: 500 µm. *p < 0.05, **p < 0.01, ***p < 0.001 between the indicated groups.

To demonstrate that Arg-II promotes mtROS production resulting in increased cancer cell malignancy indeed through suppression of Sirt3, *sirt3* was co-overexpressed with *arg-ii* in melanoma cells. *sirt3* overexpression prevented *arg-ii* overexpression-indued increase in mtROS generation (Figures 7A, B), cell number (Figures 7A, C), the percentage of cells with multi-lobed nuclei (Figures 7A, D). Moreover, Arg-II-induced DNA damage as monitored by p-γH2AX-S139 (Figures 7E, F), the increase in colony formation (Figure 8A) and migration (Figure 8B) induced by *arg-ii* overexpression were prevented by *sirt3* co-overexpression. Similar results were obtained in A549 lung cancer cells (Supplementary Figure S8). These data provide evidence that Arg-II suppresses Sirt3 leading to enhanced mtROS production, which in turn promotes melanoma growth and malignancy.

### Hypoxia suppresses Sirt3 and induces nuclear deformation by up-regulating Arg-II

**FIGURE 9:**
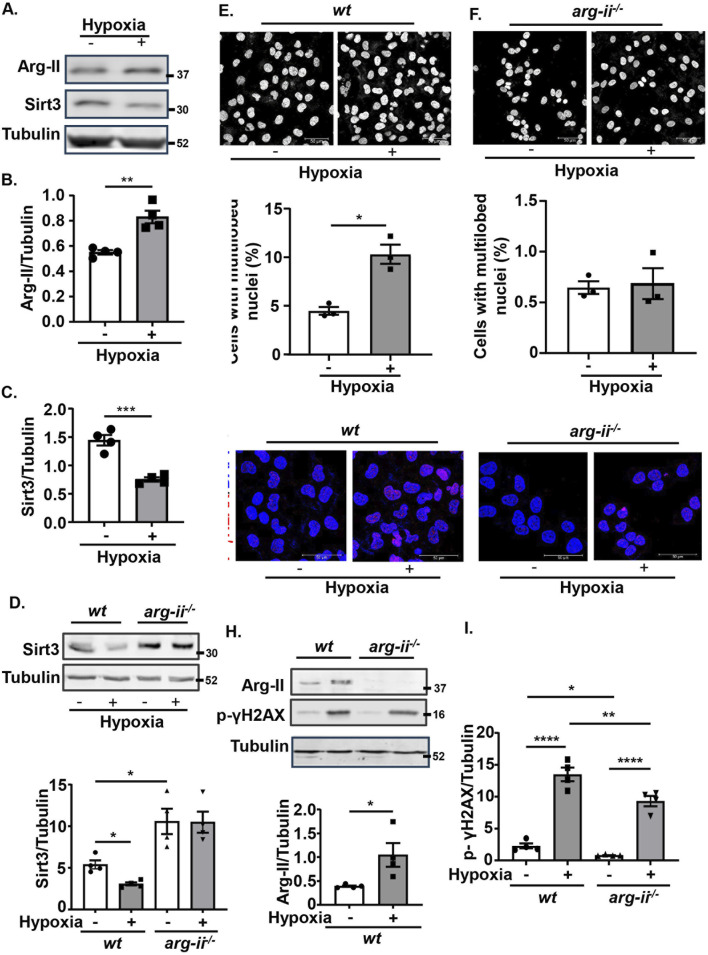
Hypoxia suppresses Sirt3 and induces nuclear deformation and DNA damage through up-regulating Arg-II. *wt* and *arg-ii* ^*−/−*^ Me276 cells were exposed to normoxia (21% O2) or hypoxia condition (1% O2) for 72 h and then subjected to Confocal microscopy cell imaging analysis and Immunoblotting analysis. **(A–C)** Immunoblotting analysis of Arg-II and Sirt3 in *wt* Me276 cells. **(D)** Immunoblotting analysis of Sirt3. **(E–F)** Immunofluorescence staining with DAPI (white). **(G)** Immunostaining for p-γH2AX-S139 (red) followed by the counter stain of nuclei with DAPI (blue). **(H, I)** Immunoblotting analysis of Arg-II, p-γH2AX-S139. Tubulin served as loading control. Scale bar: 50 µm. Quantification of the signals in images shown in **(A, D–H)** are presented as graphics in the corresponding figures (n = 4). *p < 0.05, **p < 0.01, ***p < 0.001, ****p < 0.0001.

Hypoxia is a key feature of many solid tumours and is associated with increased metastasis and poor survival in patients (Hockel and Vaupel, 2001). Given that hypoxia is a strong stimulus upregulating Arg-II in various cells (Ren et al., 2022; Liang et al., 2019; Krotova et al., 2010; Prieto et al., 2011; Chen et al., 2014; Liang et al., 2021), we wish to examine whether hypoxia enhances melanoma cell malignancy by upregulating Arg-II. As observed in the other cell types, hypoxia also enhanced Arg-II in *wt* Me276 melanoma cells, which was accompanied by a decrease in Sirt3 (Figures 9A–C), increase in nuclear deformation (Figure 9E) and DNA damage (Figure 9G). Importantly, knockout of *arg-ii* (*arg-ii* ^*−/−*^) not only enhanced Sirt3 under normoxic condition (Figure 9D), but also reduced hypoxia-induced decrease in Sirt3 (Figure 9D), increase in cells with multi-lobed nuclei (Figure 9F), DNA damage (Figures 9G–I). The results implicate that hypoxia suppresses Sirt3 and induces nuclear deformation and DNA damage through Arg-II-Sirt3 cascade.

### Elevated *arg-ii* mRNA levels in human tumor samples and is associated with poorer prognosis

To strengthen translational implications, we analysed public datasets. Analysis of the open-source database GEPIA (Gene Expression Profiling Interactive Analysis) (http://gepia.cancer-pku.cn/index.html) reveals that *arg-ii* mRNA levels are elevated in various tumor samples compared to normal counterparts, including skin cutaneous melanoma (SKCM), lung adenocarcinoma, and lung squamous cell carcinoma (Supplementary Figure S9A). Moreover, Kaplan-Meier survival curves for SKCM patients shows that higher *arg-ii* levels are associated with lower survival rate (please see the figure on website https://www.proteinatlas.org/ENSG00000081181-ARG2/cancer/melanoma#SKCM_TCGA). Similar results were observed with Kaplan-Meier survival curves for lung cancer patients of all stages obtained from the open-source website (https://kmplot.com/analysis/) (Supplementary Figure S9B). These clinical data support our findings and further imply that targeting Arg-II would be a potential therapeutic strategy for melanoma and lung cancer treatment.

## Discussion

Our present study uncovers a previously undescribed molecular mechanism of Arg-II in the control of melanoma and lung cancer cell growth and malignancy. Using both melanoma and lung carcinoma cells in culture, we delineated a previously undescribed mechanism of mtROS production by Arg-II, that is due to suppression of Sirt3 levels. This mechanism leads to enhanced cancer cell growth and migration activities, nuclear deformation and augmented DNA damage, which could potentially contribute to the cancer progression and malignancy.

It is well documented that Arg-II is implicated in various processes of cancer development and progression. Several studies have reported that Arg-II is upregulated in various cancer types, including prostate cancer, breast cancer, colon cancer, and pancreatic cancer (Zaytouni et al., 2017; Ino et al., 2013; Bronte et al., 2005; Singh et al., 2013; Porembska et al., 2003; Porembska et al., 2001). Increased arginase activity in blood is associated with poor prognosis and survival outcomes (Roci et al., 2019; Wu et al., 1994; Chrzanowska et al., 2014). In an earlier study, we showed that the mitochondrial isoform Arg-II is enhanced in human metastatic melanoma tissues as compared to non-metastatic samples and promotes melanoma cell migration and adhesion activity (Yu et al., 2020). In the current study, we further explored molecular mechanisms by which Arg-II promotes tumor cell growth and malignancy in human melanoma cells and lung cancer cells. It has been shown that mtROS plays a critical role in tumorigenesis and malignant progression (Idelchik et al., 2017) and Arg-II promotes mtROS production in various cells (Ren et al., 2022; Liang et al., 2019; Liang et al., 2021). It is presumable that Arg-II may promote tumour cell growth and malignancy via mtROS. Indeed, we demonstrate that mtROS is inhibited in *arg-ii* ^*−/−*^ and enhanced in *arg-ii*-overexpressing melanoma cells and lung cancer cells. The inhibition or enhancement of mtROS by regulating *arg-ii* gene in the tumour cells is well associated with cell growth and migration and signs of malignancy, i.e., nuclear deformation and DNA damage. Importantly, suppressing mtROS with TEMPO prevented Arg-II-promoted cancer cell growth and migration in both cancer types, demonstrating an essential role of mtROS in Arg-II-induced tumour development.

To explore how Arg-II promotes mtROS generation, we investigated the effect of a mitochondrial tumour suppressor Sirt3 that has been reported to limit tumour growth by inhibiting mtROS production (Bell et al., 2011; Haigis et al., 2012). In agreement with previous report, a tumour suppressive role of Sirt3 through mtROS is indeed demonstrated in the current study in both melanoma and lung cancer cells, whereby silencing Sirt3 causes an enhanced mtROS along with an increase in nuclear deformation, DNA damage, and cell migration and tumor colony formation. Moreover, the inhibiting effect of Arg-II on Sirt3 in both melanoma and lung cancer cells is supported by several lines of evidence. Firstly, overexpression of *arg-ii* reduces Sirt3 levels, while knockout of *arg-ii* enhances Sirt3 levels. Secondly, hypoxia upregulates Arg-II with concomitant downregulation of Sirt3, which is prevented by *arg-ii* knockout. The conclusion that Sirt3 suppression is responsible for Arg-II-induced mtROS generation is confirmed by the experiments showing that co-overexpression of *sirt3* and *arg-ii* prevented enhanced mtROS levels, cell growth, nuclear deformation, and DNA damage. These results provide evidence that Arg-II promotes mtROS through suppressing Sirt3 in the cancer cells, leading to enhanced tumour growth and malignancy. It is to mention that Arg-II-induced suppression of Sirt3 is independent of mtROS in the tumor cells, since inhibiting mtROS with TEMPO does not affect Sirt3 levels, demonstrating that there is no mutual regulation between Sirt3 and mtROS and the enhanced mtROS is due to suppression and Sirt3. The precise mechanisms by which Arg-II regulates Sirt3 remains elusive and requites further investigation. One possible mechanism could be through affecting NO production by decreasing the availability of L-arginine, the common substrate of Arg-II and NOS. NO is a signaling molecule that can regulate mitochondrial function and energy metabolism by increasing Sirt3 activity (Wang et al., 2019). However, neither iNOS, eNOS nor nNOS was detectable in the melanoma or lung carcinoma cells we tested. NOS/NO is thus unlikely the mechanism by which Arg-II modulates Sirt3. Further studies need to be done to increase our understanding of the precise mechanisms of regulation of Sirt3 by Arg-II.

Another important finding of our study is the role of Arg-II in nuclear deformation linking to enhanced migratory and invasion potential the melanoma and lung carcinoma cells, which is also mediated through the Sirt3-mtROS axis. For many years, nuclear deformation has been used by pathologists as an indication of metastatic potential (Zink et al., 2004; Nyirenda et al., 2011; Mueller et al., 2016), since nuclear deformation has been linked to cancer malignancy by causing DNA damage and promoting cancer cells migration that requires cells to deform their nucleus to squeeze through the available spaces (Kalukula et al., 2022). Here we show that overexpression of *arg-ii* or elevated expression of *arg-ii* triggered by hypoxic conditions could both induce nuclear deformation through the decreased Sirt3/increased mtROS axis in both types of cancer cells as demonstrated in our current study. In supporting the notion that multi-lobed nuclei are associated with higher migration activity (Pfeifer et al., 2019; Mierke, 2019; Das et al., 2019), our results from the trans-well migration assay showed that, compared with the control group, a greater number of *arg-ii*-overexpressing tumor cells passed diameter-restricted holes in the membrane, demonstrating that the Arg-II-mediated mtROS through suppressing Sirt3 induces nuclear deformation, leading to enhanced migration of the cancer cells. This result represents a novel mechanism for Arg-II to promote tumor metastasis and malignancy. Alterations in the nuclear envelope and cytoskeleton are believed to affect nuclei shape (Wang et al., 2018; Webster et al., 2009). In cancer cells, changes in the composition or organization of the cytoskeleton can lead to increased nuclear deformation indirectly (Lamm et al., 2020). Our previous study has shown an involvement of a cytoskeleton protein in Arg-II’s biological effect, i.e., myosin 1b (Myo1b) mediates the effect of Arg-II in activating mTORC1-S6K1 through promoting peripheral lysosomal positioning (Yu et al., 2018). Given that excessive contractile myosin-II activity has been reported to be necessary for abnormal nuclear morphologies and chromatin disorganization in Cof/ADF silenced cells (Wiggan et al., 2017), a pressing goal for the future will be to investigate whether Arg-II-Sirt3-mtROS axis promotes nuclear deformation in cancer cells through Myo1b and/or other cytoskeleton proteins.

DNA damage is a common feature of cancer cells, and it contributes to the development and progression of many cancers (Lord and Ashworth, 2012). ROS is a well-known inducer of DNA damage leading to the genomic instability in cancers (Renaudin, 2021) linking to cancer malignancy and therapy resistance (Ui et al., 2020; Reislander et al., 2020). Growing evidence has also been presented that nuclear deformation causes DNA damage (Shah et al., 2021). In supporting this notion, we show here that Arg-II indeed induces DNA damage as reflected by enhanced p-γH2AX levels through decreased Sirt3/increased mtROS axis in both cancer cell types. It is well documented that exposure of cells to extracellular ROS (such as H_2_O_2_) triggers the DNA damage and H_2_O_2_ (via •OH) oxidizes DNA. As mitochondria are one of the major intracellular sources of ROS, it is believed that ROS production and release by mitochondria leads to oxidative DNA damage and mutation. Our data in the current study also demonstrate that elevated Arg-II or silencing *sirt3* promoted mitochondrial ROS (mtROS) with concomitant DNA damage, which was reversed by suppressing mtROS using antioxidant TEMPO, supporting the notion that overproduction of mtROS contributes to tumorigenicity by induction of genomic instability. However, recent evidence suggests that local mtROS generation does not reach the nucleus (Hoehne et al., 2022; van Soest et al., 2024), whereas DNA-base oxidation by mtROS could only occur in case that mtROS would diffuse into the nucleus. Thus, it is unlikely that mtROS leads to genomic instability by its direct effects. It remains unclear to what extent respiration-derived ROS contribute directly to DNA damage (van Soest et al., 2024).

In addition to the genetic approaches of *arg-ii* overexpression and knockout or silencing, the major conclusions of the current study are also confirmed in both cancer cell types under hypoxia conditions. Hypoxia is a common feature of many solid tumours (Yasuda, 2008), is a strong stimulus for upregulating Arg-II in different cell types (Ren et al., 2022; Liang et al., 2019; Krotova et al., 2010; Prieto et al., 2011; Chen et al., 2014; Liang et al., 2021; Krause et al., 2012; Banerjee et al., 2021). Remarkably, hypoxia enhances expression of Arg-II in melanoma cells with concomitant suppression of Sirt3 and augmented mtROS along with more deformed nuclei and DNA damage. All these features are effectively prevented by knocking out *arg-ii*. These data imply that targeting Arg-II in hypoxic cancers may be a potential therapeutic strategy.

Previous studies showed that Arg-II can promote melanoma metastasis and adhesion to endothelial cells by activating the STAT3 pathway (Yu et al., 2020). In the current study, we demonstrate another mechanism by which Arg-II promotes melanoma growth and metastasis-related nuclei deformation and migration. Sirt3 has been shown to deacetylate STAT3 (Guo et al., 2017), which results in the inhibition of its activation and signaling. This deacetylation event also promotes the ubiquitination and degradation of STAT3, further inhibiting its activity (Wei et al., 2018; Wu et al., 2023). Moreover, other studies showed that Sirt3-STAT3 could work as a regulatory axis, which may activate and affect inflammation and neurodegenerative diseases regulated by AMPK signaling (Zhou et al., 2023; Hu et al., 2023). It is thus tempting to speculate that Arg-II activates STAT3 through suppressing Sirt3. Whether Arg-II-Sirt3 regulates mtROS and nuclear deformation through activating the STAT3 signal, or whether the STAT3 signaling is also modulated by mtROS require further investigation.

**FIGURE 10:**
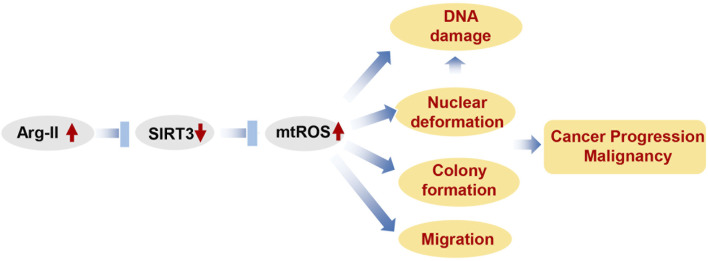
Schematic summary of how Arg-II promotes cancer progression. Elevated Arg-II in cancer cells suppresses Sirt3 leading to enhanced mtROS production. mtROS promotes cancer growth, DNA damage and nucelear deformation. Nuclear deformation in turn promotes migration as well as inducing DAN damage. All these contribute to cancer progression and malignancy.

The data presented here are summarized in a scheme that depicts our view of how Arg-II promotes cancer progression (Figure 10). Our study demonstrates that Arg-II promotes cancer progression including growth and malignancy such as metastasis-related process migration and DNA damages through a newly uncovered mechanism, i.e., suppression of Sirt3/enhancement of mtROS axis. These mechanisms are involved in hypoxia-promoted malignancy of cancer, which could indirectly lead to resistance to treatment. It is worth noting, our finding is supported by patient-derived data from publicly accessible datasets showing higher arg-ii levels in several cancers including melanoma and lung cancers associate poorer survival rate, which strengthens translational implications of our study. It opens a new window for future research on targeted anticancer drugs, especially different solid tumors under hypoxic conditions in which Arg-II could be upregulated. Targeting Arg-II would be a potential therapeutic strategy for melanoma and lung cancer treatment.

## Data Availability

The original contributions presented in the study are included in the article/Supplementary Material, further inquiries can be directed to the corresponding author.

## Abbreviations

Arg-II/*arg-ii*, Arginase-II/*arginase-ii*; MOI, Multiplicity of infection; mtROS, Mitochondrial reactive oxygen species; NBF, Neutral buffered formalin; NO, Nitric oxide; NOS, Nitric oxide synthase; SDS-PAGE, Sodium dodecyl sulphate-polyacrylamide gel; siRNA, Small interfering RNA; Sirt3, Sirtuin 3; WGA, Wheat germ agglutinin.
