# Supplementary material for: Arginase-II promotes melanoma and lung cancer cell growth by regulating Sirt3-mtROS axis
Source: Front Cell Dev Biol. 2025 Mar 19;13:1528972. doi: 10.3389/fcell.2025.1528972 (PMC11961885; doi:10.3389/fcell.2025.1528972)
Supplement: Supplementary file 1 [file Table1.pdf]

Supplemental Table 1. Dilutions of antibody

Arg-II 1st antibody	1:1000 for WB
SIRT3 1st antibody	1:1000 for WB
SIRT1 1st antibody	1:1000 for WB
p-gH2AX antibody	1:5000 for WB, 1: 1000 for IF
Lamin B1 1st antibody	1:1000 for WB
Lamin A/C 1st antibody	1:1000 for WB
Tubulin 1st antibody	1:5000 for WB
Goat anti-rabbit 2nd antibody	1:5000 for WB
Goat anti-mice 2nd antibody	1:5000 for WB
