# Supplementary material for: Arginase-II promotes melanoma and lung cancer cell growth by regulating Sirt3-mtROS axis
Source: Front Cell Dev Biol. 2025 Mar 19;13:1528972. doi: 10.3389/fcell.2025.1528972 (PMC11961885; doi:10.3389/fcell.2025.1528972)
Supplement: Supplementary file 2 [file DataSheet1.pdf]

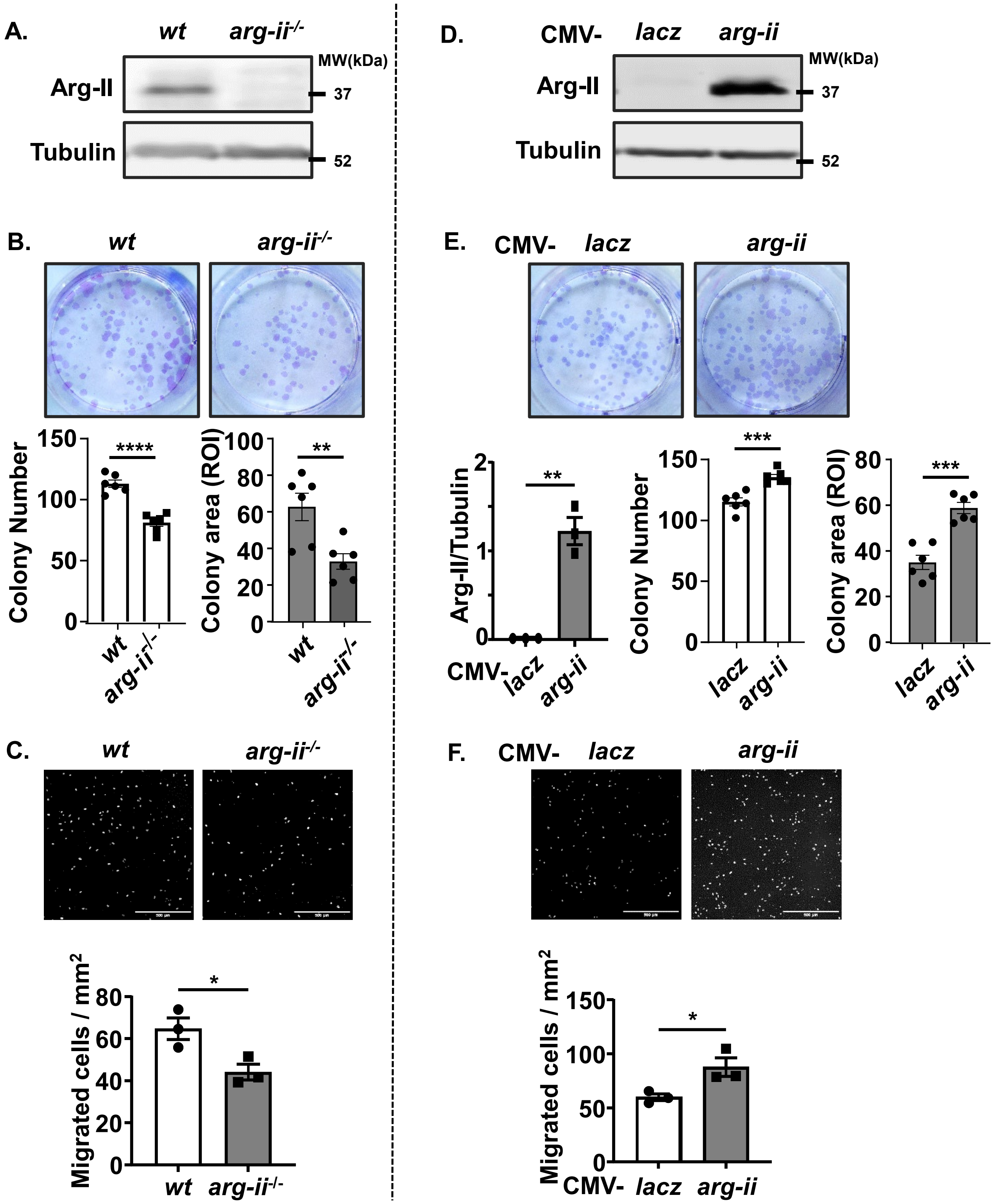

Fig. S1. Arg-II promotes colony formation and migration of A549 lung carcinoma cells. Experiments were performed as described in Fig.1, except that A549 lung carcinoma cells instead of Me276 melanoma cells were used. (**A & D**) Immunoblotting analysis. (**B & E**) Colony formation assay. ROI: region of interest. (**C & F**) The trans-well migration assay. The graphics present the quantification of signals, colony numbers and area, migrated cell numbers of the corresponding images. Scale bar: 500 µm. *$p < 0.05$, **$p < 0.01$, ***$p<0.001$. ****$p<0.0001$

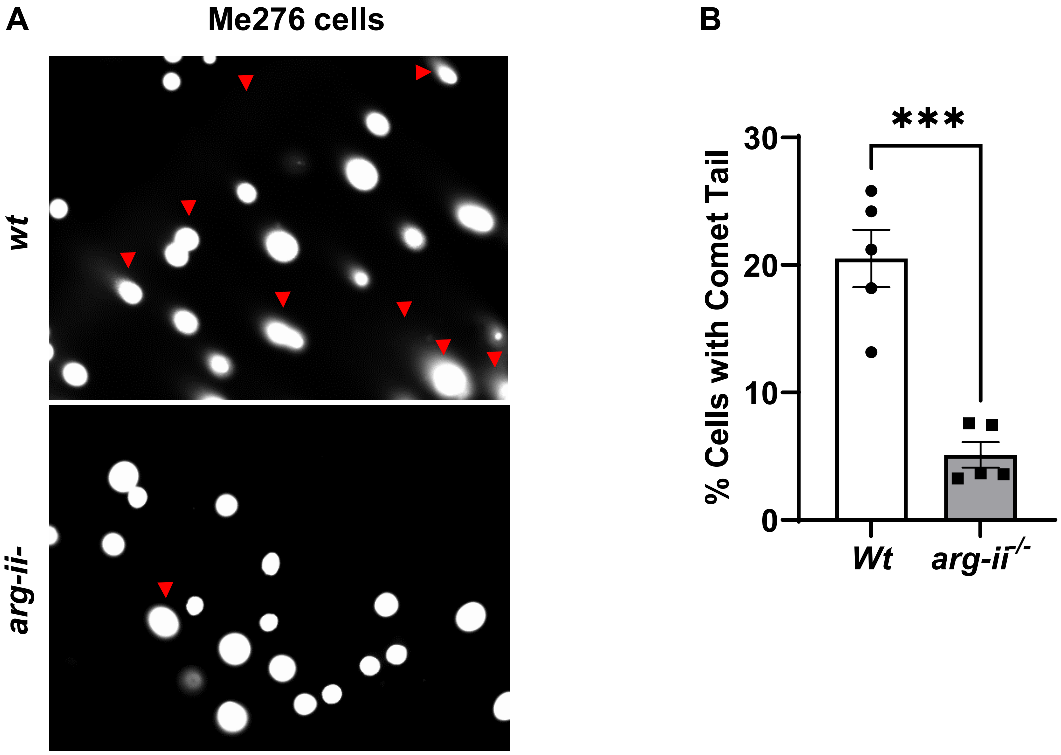

Fig. S2. Arg-II knockout reduces the number of cells with DNA damage. (A) A comet assay was performed as described in the Methods section. Wild-type (wt) and *arg-ii*$^{-/-}$ Me276 melanoma cells were seeded for 24 hours, and the assay was performed according to the manufacturer's instructions. Representative images from four biological replicates are shown. Red arrows indicate cells positive for comet tails, a marker of DNA damage. **(B)** The percentage of comet tail-positive cells was quantified from four independent trials and represented as the percentage of total cells. The ***p < 0.001.

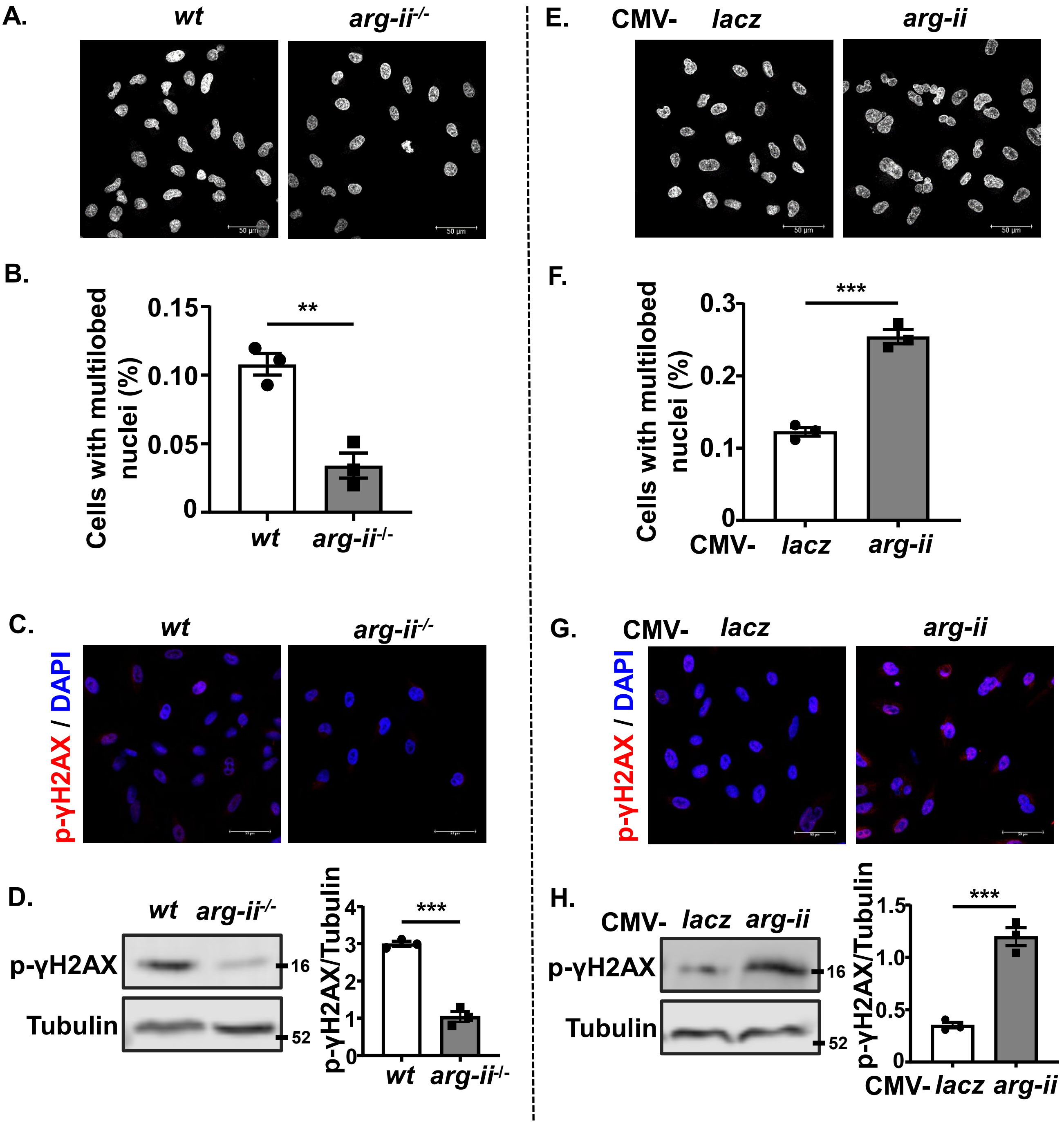

Fig. S3. Arg-II over-expression induces the nuclear deformation and DNA damage in A549 lung cancer cells. Experiments were performed as described in Fig. 2, except that A549 instead of Me276 cells were used. (**A & E**) Confocal microscopic images of nuclei staining with DAPI (white). (**B & F**) Quantification of numbers (presented as %) of cells with multilobed nuclei shown in A & E, respectively. (**C & G**) Immunofluorescence staining for p-γH2AX (red) followed by counter stain of nuclei with DAPI (blue). (**D & H**) Immunoblotting analysis of p-γH2AX, tubulin was used as loading control. Scale bar: 50 µm. The graphics present the quantification of signals shown in the corresponding images. n=3. $^{**}p < 0.01$, $^{***}p < 0.001$.

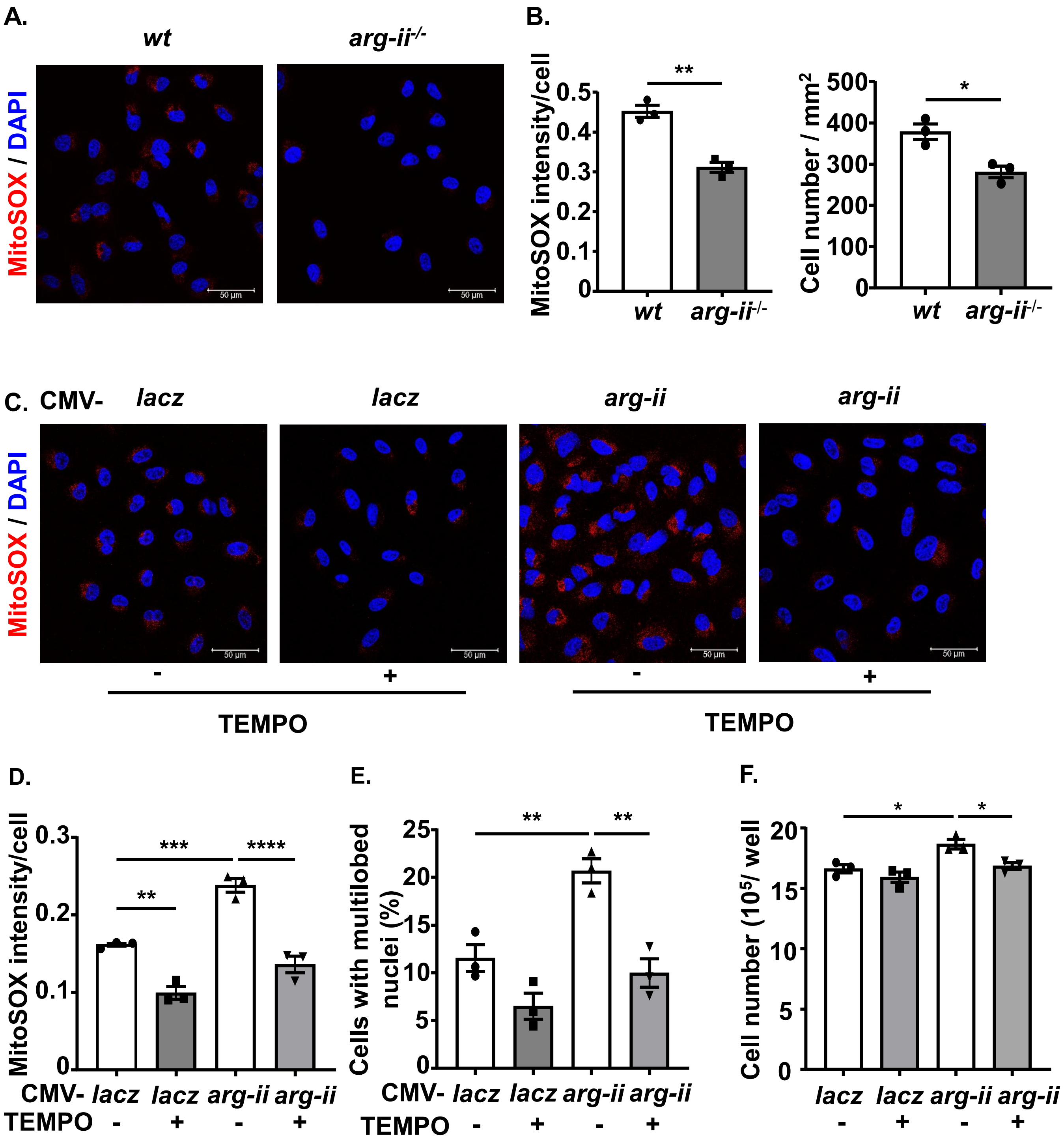

Fig. S4. Arg-II promotes A549 lung carcinoma cell growth and nuclear deformation via mtROS. Experiments were performed as described in Fig. 3, except that A549 instead of Me276 cells were used. (**A & C**) Confocal microscopic images of mtROS staining with MitoSOX (red) and DAPI (blue). (**B**) The graphics present the quantification of the MitoSOX signal and cell number per mm^2 in the images shown in A. (**D & E**) The graphics present quantification of images shown in C for MitoSOX and % of cells with multilobed nuclei as indicated. **(F)** Cell number per well counted with the Neubauer cell counting chamber. Scale bar: 50 µm. *$p < 0.05$, **$p < 0.01$, ***$p<0.001$. ****$p<0.0001$.

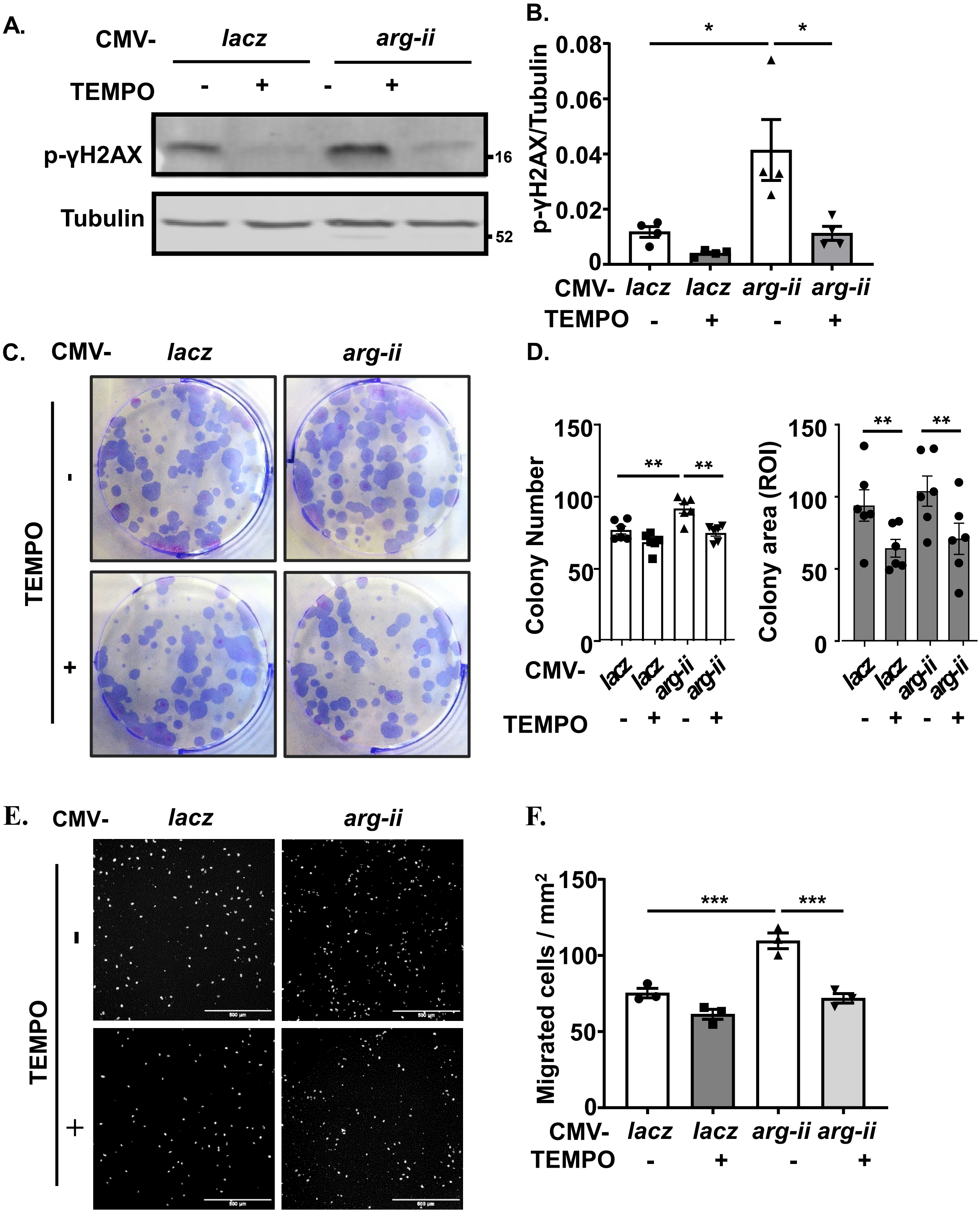

Fig. S5. Arg-II promotes DNA damage, colony formation and migration through mtROS in A549 lung carcinama cells. Experiments were performed as described in Fig. 3 & 4, except that A549 instead of Me276 cells were used. **(A & B)** Immunoblotting analysis of p-γH2AX. Tubulin served as loading control. n=4. **(C & D)** Colony formation assay (n=6). ROI: region of interest. **(E & F)** Trans-well migration assay. n=3. Scale bar: 500 µm. $*p < 0.05$, $**p < 0.01$, $***p < 0.001$.

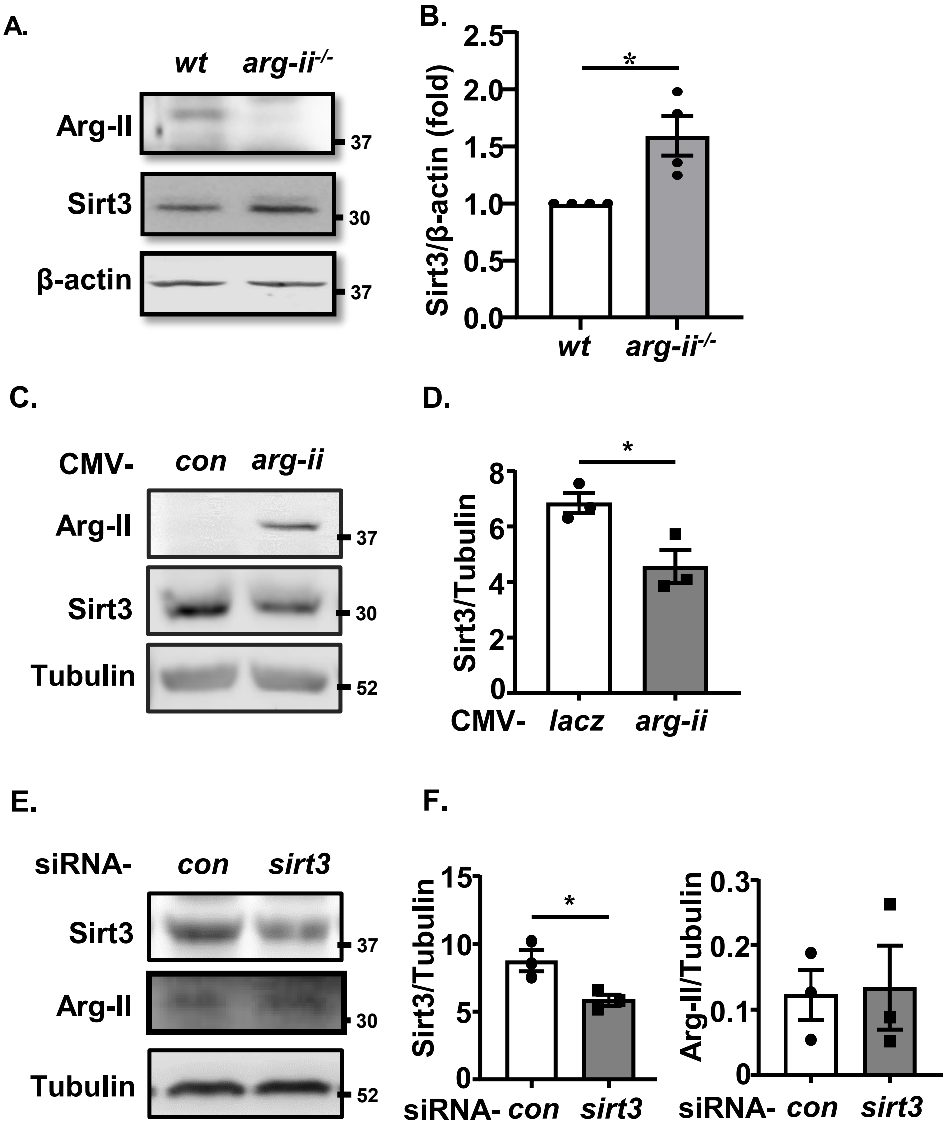

Fig. S6. Arg-II suppresses SIRT3 expression in A549 lung carcinoma cells. Experiments were performed as described in Fig. 5 except that A549 instead of Me276 cells were used. The graphics on the right present the quantification of the results shown in the corresponding left panels. n=3. *$p < 0.05$,

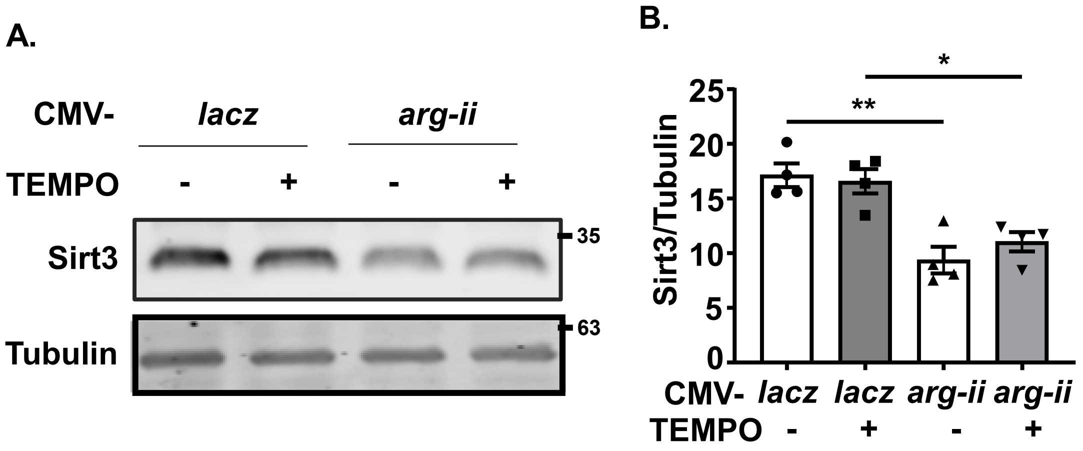

Fig. S7. mtROS does not mediate Arg-II-induced suppression of SIRT3 in Me276 melanoma cells. *wt* Me276 melanoma cells were transfected with rAd-CMV-*lacz* as control or rAd-CMV-*arg-ii* to overexpress Arg-II. 48h post transduction, cells were treated with TEMPO (10 µmol/L) 1h and then removed. Cells were harvested after further cultivation for 24 h and subjected to immunoblotiing analysis. (**A**) Immunoblotting analysis. (**B)** Quantification of the signals shown in A. n=4. *$p < 0.05$, **$p < 0.01$.

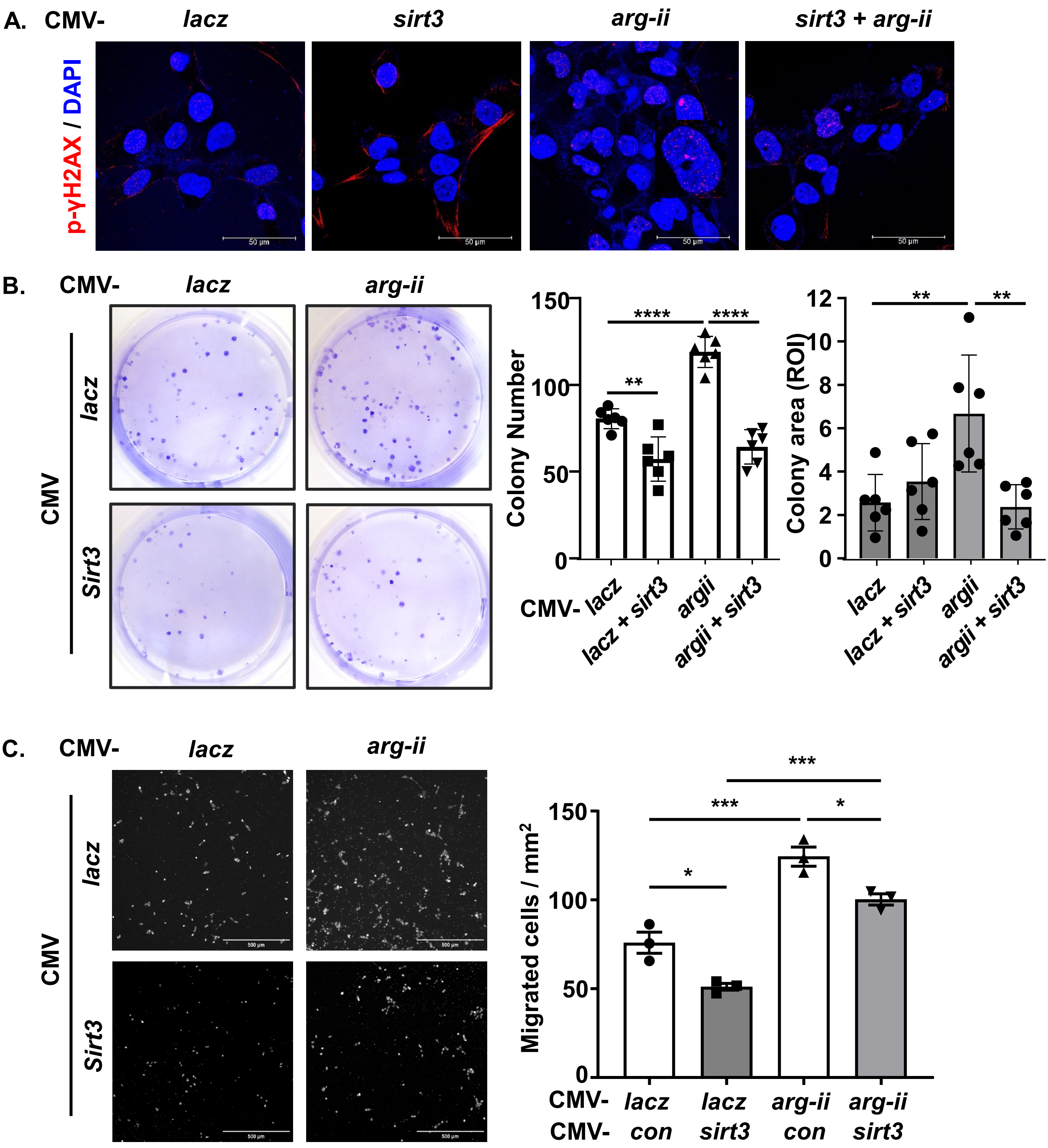

Fig. S8. Sirt3 prevents Arg-II-induced DNA damage, colony formation and migration in A549 lung carcinoma cells. Experiments were performed as described in Fig. 7 and 8, except that A549 instead of Me276 cells were used. **(A)** Confocal microscopic images of immunofluorescence staining for p-γH2AX (red) and DAPI staining for nuclei (blue). Scale bar: 50 µm. **(B)** Colony formation assay (n=6). ROI: region of interest. **(C)** Trans-well migration assay. n=3. Scale bar: 500 µm. *$p < 0.05$, **$p < 0.01$, ***$p < 0.001$, ****$p < 0.0001$.

A

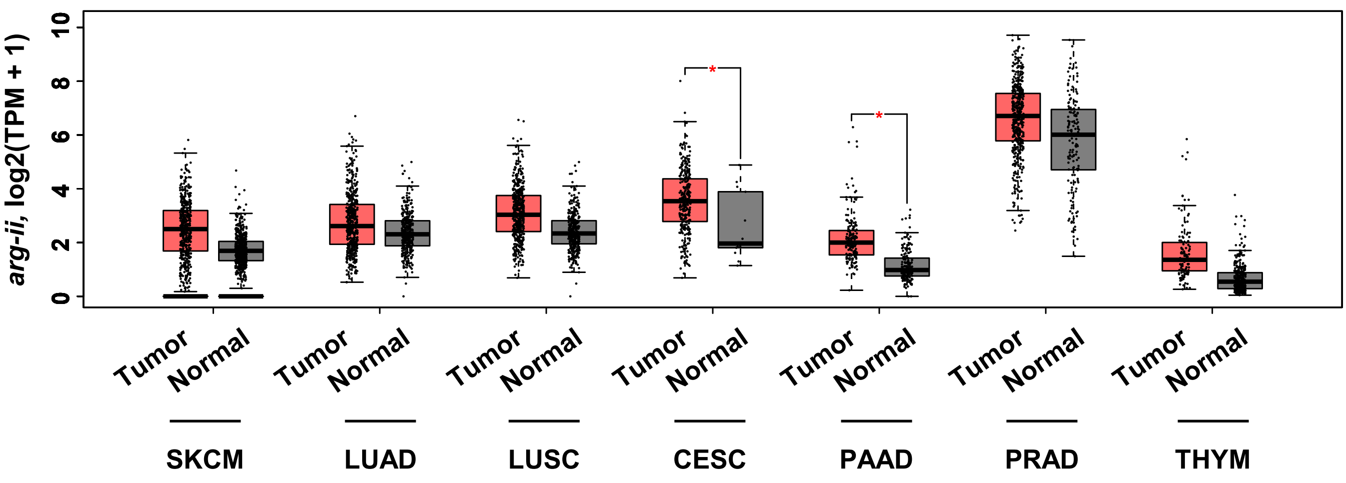

B

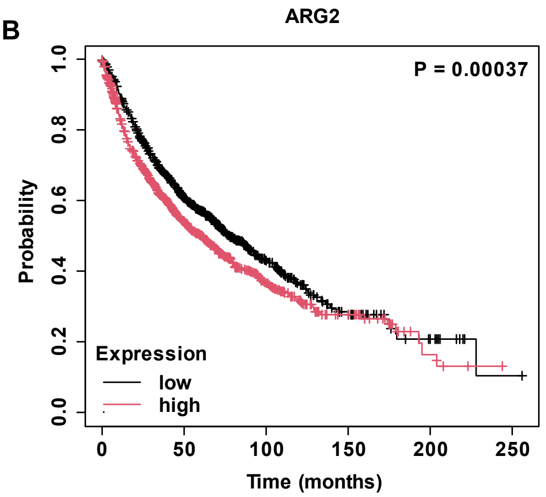

Fig. S9. Elevated *arg-ii* mRNA levels in human tumor samples. (A) Gene expression datasets from the GEPIA website (*http://gepia.cancer-pku.cn/index.html*) were analyzed for *arg-ii* mRNA levels in various cancers: SKCM (Normal: n=558, Tumor: n=461), LUAD (Normal: n=347, Tumor: n=483), LUSC (Normal: n=338, Tumor: n=486), CESC (Normal: n=13, Tumor: n=306), PAAD (Normal: n=171, Tumor: n=179), PRAD (Normal: n=152, Tumor: n=492) and THYM (Normal: n=339, Tumor: n=118). Elevated Arg-II gene expression was observed in tumor samples compared to normal counterparts. **(B)** Kaplan-Meier survival curves for all stage lung cancer patients were obtained from the open-source website Kmplot (*https://kmplot.com/analysis/*). SKCM, Skin cutaneous melanoma; LUAD, Lung adenocarcinoma; LUSC, Lung squamous cell carcinoma; CESC, Cervical squamous cell carcinoma and endocervical adenocarcinoma; PAAD, Pancreatic adenocarcinoma; PRAD, Prostate adenocarcinoma; THYM, Thymoma.
